# miR-1827 inhibits osteogenic differentiation by targeting IGF1 in MSMSCs

**DOI:** 10.1038/srep46136

**Published:** 2017-04-07

**Authors:** ShuangXi Zhu, Wei Peng, Xiang Li, JunQuan Weng, Xing Zhang, JunBing Guo, DaiYing Huang, Qiong Rong, SongLing Chen

**Affiliations:** 1 Department of Oral and Maxillofacial Surgery, the First Affiliated Hospital, Sun Yat-sen University, Guangzhou, PR China; 2 Guang dong Key Laboratory of Stomatology, Sun Yat-sen University, Guangzhou, PR China; 3 Department of Stomatology, Guangdong Provincial Hospital of Traditional Chinese Medicine, Guangzhou, PR China.; 4 Department of Prosthetic Dentistry, the First People’s Hospital of Yunnan, Kunming, PR China

## Abstract

We recently reported that maxillary sinus membrane stem cells (MSMSCs) have osteogenic potential. However, the biological mechanisms of bone formation remain unclear. In this study, we investigated the role and mechanisms of microRNAs (miRNAs) in the osteogenic differentiation of MSMSCs. The expression of miRNAs was determined in differentiated MSMSCs by comprehensive miRNA microarray analysis and quantitative RT-PCR (qRT-PCR). We selected miR-1827 for functional follow-up studies to explore its significance in MSMSCs. Here, miR-1827 was found to be up-regulated during osteogenic differentiation of MSMSCs. Over expression of miR-1827 inhibited osteogenic differentiation of MSMSCs *in vitro*, whereas the repression of miR-1827 greatly promoted cell differentiation. Further experiments confirmed that insulin-like growth factor 1 (IGF1) is a direct target of miR-1827. miR-1827 inhibited osteogenic differentiation partially via IGF1, which in turn is a positive regulator of osteogenic differentiation. Moreover, miR-1827 suppressed ectopic bone formation and silencing of miR-1827 led to increased bone formation *in vivo.* In summary, this study is the first to demonstrate that miR-1827 can regulate osteogenic differentiation. The increase in miR-1827 expression observed during osteogenesis is likely a negative feedback mechanism, thus offering a potential therapeutic target to address inadequate bone volume for dental implantation through inhibiting miR-1827.

Maxillary sinus floor elevation is an effective and widely used technique for augmenting alveolar bone insufficiency in the posterior maxilla before dental implant placement^1^,^2^. Recent findings have shown that the maxillary sinus membrane possesses osteogenic potential. Osteoprogenitor cells harvested from the maxillary sinus membrane have osteogenic potential both *in vitro and in vivo*^3^,^4^,^5^. We have previously demonstrated that human maxillary sinus membrane stem cells (MSMSCs) have osteogenic differentiation potential *in vitro*^6^. In addition, MSMSCs participate in bone formation in nude mice and can retain their stem cell-like properties after long-term *in vivo* transplantation^6^. Nevertheless, the molecular mechanisms of bone formation remain poorly understood.

MicroRNAs (miRNAs) are small non-coding RNAs that play an important role in gene regulation^7^,^8^. miRNAs inhibit gene translation and/or target them for cleavage and degradation by binding to target mRNAs. miRNA binding sites are generally located in the 3′-untranslated regions (3′-UTRs) of target mRNAs^7^,^8^,^9^. Bioinformatics studies have demonstrated that miRNAs may regulate one-third of the transcriptome, indicating the essential role of miRNAs in regulating gene expression^10^. Accumulating evidence shows that miRNAs participate in the control of osteogenic differentiation^11^,^12^. For example, heparin-binding epidermal growth factor-like growth factor (HB-EGF) is a negative regulatory factor of osteogenic differentiation. miR-96 stimulates osteogenic differentiation by directly targeting the 3′-UTR of HB-EGF^13^. In addition, miR-194 can suppress adipogenesis and enhance osteogenesis by inhibiting chicken ovalbumin upstream promoter-transcription factor II (COUP-TFII)^14^. Furthermore, over expression of miR-204 and miR-637 was discovered to promote adipocyte differentiation and inhibit osteogenic differentiation by inhibiting runt-related transcription factor 2 (Runx2) or Osterix (Osx)^15^,^16^. These findings demonstrate that miRNAs significantly impact osteogenic differentiation and bone formation. However, the role of miRNAs in the osteogenic differentiation of MSMSCs remains unclear.

Herein, miR-1827 was found to be up-regulated during osteogenic differentiation of MSMSCs and an inhibitor of cell differentiation. In addition, insulin-like growth factor 1 (IGF1), a positive regulator of osteogenesis, is a direct target of miR-1827. Although miR-1827 has been proven to play a significant role in cancer^17^,^18^, we confirmed its regulatory effect on osteogenic differentiation. This study describes a novel mechanism of osteogenic differentiation and may provide a potential therapeutic target for bone augmentation in the posterior maxilla.

## Results

### Expression of miRNAs in differentiated MSMSCs

**Figure 1:**
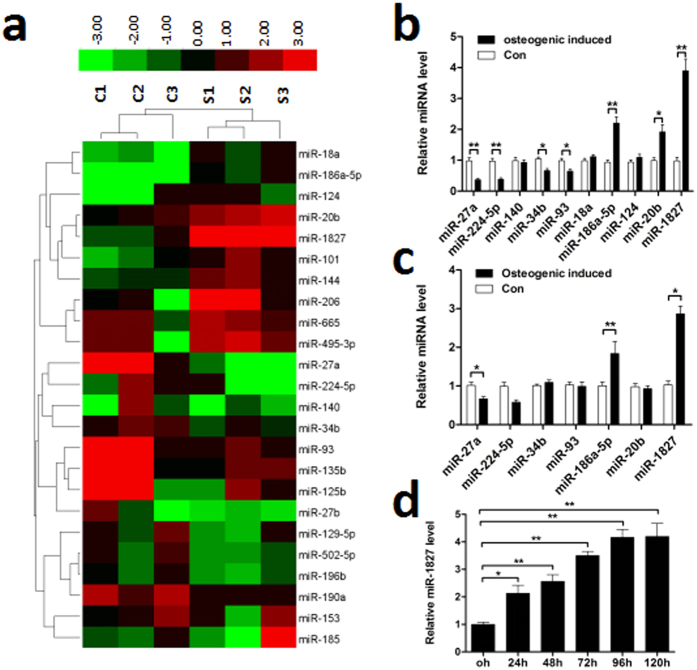
Up-regulation of miR-1827 in both differentiated MSMSCs and differentiated BMSSCs. (**a**) Alterations of miRNA expression in both differentiated and undifferentiated MSMSCs examined by Agilent miRNA arrays. Green represents down-regulated miRNAs and red represents up-regulated miRNAs, with the color scale in the upper-right corner indicating the relative expression levels. (**b**) qRT-PCR analysis of miR-27a, miR-224-5p, miR-140, miR-34b, miR-93, miR-18a, miR-186a-5p, miR-124, miR-20b and miR-1827 levels selected from the array data in MSMSCs cultured in osteogenic induction medium for 3 d (normalized to internal reference gene U6). (**c**) Expression levels of miR-27a, miR-224-5p, miR-34b, miR-93, miR-186a-5p, miR-20b and miR-1827 in differentiated BMSSCs examined by qRT-PCR. (**d**) Time course of miR-1827 expression in BMSSCs during osteogenic differentiation. For each group, values are the mean ± SD; n = 3, *P < 0.05, **P < 0.01.

miRNA expression was determined through comprehensive miRNA microarray analysis in MSMSCs cultured in osteogenic induction medium for 3 d. Several miRNAs of the ~1000 miRNA molecules on the chip were detected. Among the detected miRNAs, the expression levels of 24 miRNAs were significantly changed, with 14 miRNAs down-regulated and 10 miRNAs up-regulated compared with undifferentiated cells (the control group) (Fig. 1a). According to the fold changes of 24 miRNAs, 10 miRNAs were selected for identification by quantitative RT-PCR (qRT-PCR). The results showed that 7 miRNAs were significantly changed, with 4 miRNAs (miR-27a, miR-224-5p, miR-34b and miR-93) down-regulated and 3 miRNAs (miR-186a-5p, miR-20b and miR-1827) up-regulated (Fig. 1b).

### miR-1827 expression is up-regulated in differentiated bone marrow stromal stem cells (BMSSCs)

To further validate whether the 7 miRNAs identified by qRT-PCR vary similarly in other stem cells during osteogenic differentiation, BMSSCs were cultured in osteogenic induction medium for 3 d. The qRT-PCR results showed that only 3 miRNAs (miR-1827, miR-186a-5p and miR-27a) were significantly altered (Fig. 1c) and showed similar variation in MSMSCs. Further study demonstrated that the expression of miR-1827 increased in BMSSCs in a time-dependent manner after culture in osteogenic induction medium for at least 120 h (Fig. 1d). Notably, the role of miR-1827 in osteogenic differentiation has not yet been studied. Therefore, we selected miR-1827 for follow-up studies to explore the function of this molecule in osteogenic differentiation.

### miR-1827 inhibits osteogenic differentiation *i**n vitro***

**Figure 2:**
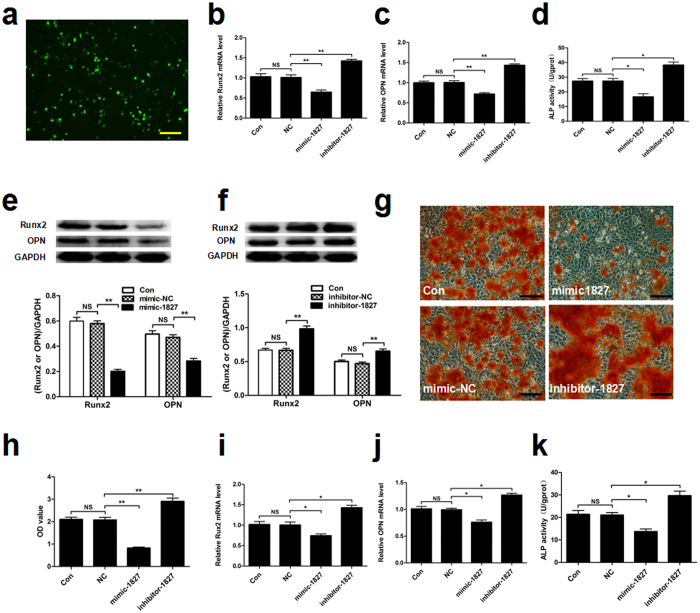
miR-1827 inhibits osteogenic differentiation *in vitro*. To evaluate the effects of miR-1827 on osteogenic differentiation, MSMSCs and BMSSCs were transfected with a miR-1827 mimic (mimic-1827), miR-1827 inhibitor (inhibitor-1827) or their respective negative controls (mimic-NC, inhibitor-NC). (**a**) Representative fluorescent images of MSMSCs transfected with a miRNA nucleoside analogue for 24 h. Fluorescence indicated transfected cells. (**b,c**) qRT-PCR analysis of osteoblastic marker (Runx2 and OPN) mRNA expression after 48 h of osteogenic induction. The gene expression levels in MSMSCs transfected with the respective miRNA negative controls were set as the control (normalized to GAPDH). (**d**) ALP activity in MSMSCs at 48 h. (**e,f)** Western blot analysis of Runx2 and OPN protein expression in MSMSCs after 48 h of osteogenic induction. GAPDH was used to assess the amount of protein loaded per sample. (**g**) The formation of mineralized nodules in MSMSCs was observed by Alizarin Red staining. Cells were cultured in osteogenic induction medium for 4 weeks. (**h**) The mineralized nodules in different groups were quantified using cetylpyridinium chloride. OD, optical density. (**i,j,k**) Alterations in osteoblastic marker (Runx2 and OPN) mRNA expression and ALP activity were observed in BMSSCs by qRT-PCR and an ALP activity assay. For each group, values are the mean ± SD; n = 3, *P < 0.05, **P < 0.01. NS, not significant. Scar bars represent 100 μm.

To study the biological role of miR-1827 in osteogenic differentiation, MSMSCs were transfected with a miR-1827 inhibitor or miR-1827 mimic to alter the expression levels of miR-1827 *in vitro* (Fig. 2a). The effects of miR-1827 inhibitor or mimic on osteogenic differentiation were evaluated by observing mineralized nodule formation, alkaline phosphatase (ALP) activity and the expression levels of Runx2 and osteopontin (OPN). After transfection with miR-1827 mimic, the mRNA expression levels of osteogenic-specific markers (Runx2 and OPN) decreased compared with the control group as indicated by qRT-PCR (Fig. 2b,c). In addition, ALP activity and the protein expression levels of Runx2 and OPN decreased (Fig. 2d,e,f). Furthermore, the formation of mineralized nodules was also reduced (Fig. 2g,h), implying that over expression of miR-1827 inhibited osteogenic differentiation. On the contrary, Runx2 and OPN mRNA expression (Fig. 2b,c), ALP activity (Fig. 2d), and protein levels of Runx2 and OPN (Fig. 2e,f) were all increased following transfection with the miR-1827 inhibitor. Furthermore, the formation of mineralized nodules was also increased (Fig. 2g,h), implying that the repression of miR-1827 promoted osteogenic differentiation. To further validate the role of miR-1827 in osteogenic differentiation, the miR-1827 mimic and inhibitor were transfected into BMSSCs. After 48 h of osteogenic induction, alterations in Runx2 and OPN mRNA expression and ALP activity were observed to be similar to those detected in MSMSCs, as indicated by qRT-PCR and an ALP activity assay (Fig. 2i,j,k). These results indicate that miR-1827 inhibits osteogenic differentiation.

### IGF1 is a target of miR-1827

**Figure 3:**
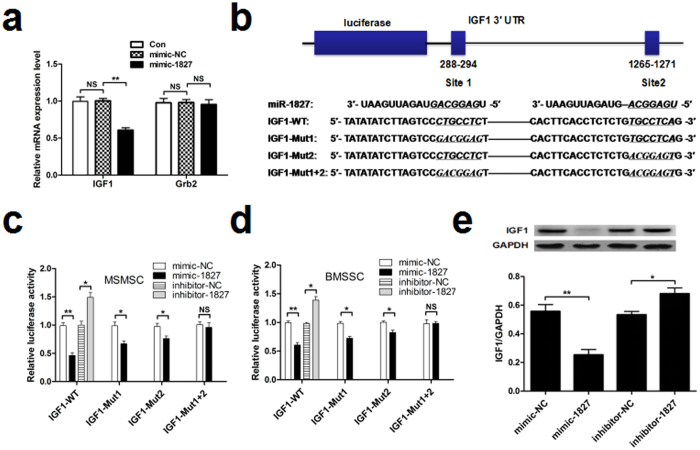
IGF1 is a target of miR-1827 in MSMSCs and BMSSCs. qRT-PCR analysis of candidate gene (IGF1 and Grb2) expression in MSMSCs after transfection with the miR-1827 mimic or negative control for 48 h. (**b**) A schematic of the luciferase reporter containing the putative binding sites for miR-1827 in the IGF1 3′-UTR. The sequences of miR-1827, its putative binding sites, and their respective IGF1 3′-UTR mutants are presented. The positions of putative binding sites are labeled. (**c,d**) Dual luciferase activity in transfected MSMSCs and BMSSCs. (**e**) Western blot analysis of IGF1 protein levels in MSMSCs after treatment with miR-1827 mimic, miR-1827 inhibitor or their respective negative controls for 48 h. For each group, values are the mean ± SD; n = 3, *P** < **0.05, **P** < **0.01. NS, not significant.

To further explore the mechanism by which miR-1827 regulates osteogenic differentiation, we examined the predicted targets of miR-1827 using TargetScan, miRanda and miRDB software. Based on these analyses, several potential targets meeting this criterion were identified (Supplementary Figure 1). From these genes, we selected insulin-like growth factor 1 (IGF1) and growth factor receptor binding protein 2 (Grb2) for further study, because these proteins are potentially involved in osteogenic differentiation through MAPK signaling pathways^19^,^20^. Therefore, we examined the mRNA expression of each target in response to the miR-1827 mimic and miR-1827 inhibitor. We observed that the mRNA expression of IGF1, but not Grb2, was significantly changed (Fig. 3a). Therefore, IGF1 was selected as the better candidate between the two genes.

Computational analysis using TargetScan and miRDB predicted that the 3′-UTRof the human IGF1 mRNA has two putative binding sites for miR-1827 within the first 1.3 kb (Fig. 3b). Thus, to identify the miR-1827 target region in IGF1 mRNA, a luciferase assay was employed. We constructed an IGF1 3′-UTR luciferase reporter containing both miR-1827 binding sites. This reporter was co-transfected with miR-1827oligos into MSMSCs and BMSSCs. The luciferase reporter assay demonstrated that the miR-1827 mimic decreased IGF1-WT 3′-UTR luciferase reporter activity in both cell lines, whereas the miR-1827 inhibitor increased IGF1-WT 3′-UTR luciferase reporter activity (Fig. 3c,d). While mutating any one of the two putative binding sites individually only partially abolished the repression induced by miR-1827, mutating both binding sites simultaneously almost completely abolished miR-1827-induced repression (Fig. 3c,d), indicating that both predicted sequences are functional miR-1827 binding sites. We also examined the protein expression levels of IGF1 in response to miR-1827. We observed that IGF1 protein levels were significantly decreased by treatment with the miR-1827 mimic and increased by treatment with the miR-1827 inhibitor (Fig. 3e). Together, these results indicated that miR-1827 targets IGF1 through direct binding to the two binding sites in the IGF1 3′-UTR.

### IGF1 promotes osteogenic differentiation of MSMSCs *in vitro*

**Figure 4:**
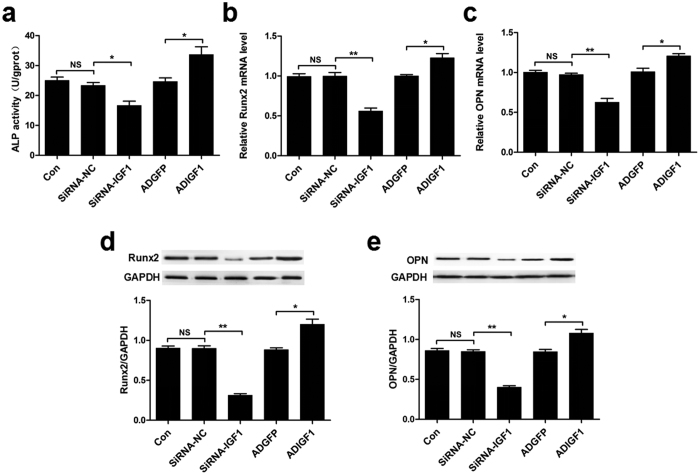
IGF1 promotes osteogenic differentiation of MSMSCs *in vitro*. (**a**) Analysis of ALP activity in MSMSCs after treatment with siRNA-IGF1, ADIGF1 or their respective negative controls for 48 h. (**b,c**) qRT-PCR analysis of Runx2 and OPN mRNA expression in MSMSCs after treatment with siRNA-IGF1, ADIGF1 or their respective negative controls for 48 h. (**d,e**) Western blot analysis of Runx2 and OPN protein expression in MSMSCs after treatment with siRNA-IGF1, ADIGF1 or their respective negative controls for 48 h. For each group, values are the mean ± SD; n = 3, *P < 0.05, **P < 0.01. NS, not significant.

To characterize the role of IGF1 in the osteogenic differentiation of MSMSCs, we used siRNA and recombinant adenoviruses to study the effects of IGF1 loss- and gain-of-function. The regulative effect of siRNA-IGF1 and recombinant adenoviruses expressing IGF1 (ADIGF1) on IGF1 protein expression were shown in Supplementary Fig. 2. An ALP activity assay and qRT-PCR results showed that transfection with siRNA-IGF1 markedly decreased ALP activity and mRNA expression levels of Runx2 and OPN compared to transfection with siRNA-NC (Fig. 4a,b,c). Infection with IGF1 (ADIGF1) significantly increased ALP activity and mRNA expression levels of Runx2 and OPN compared to infection with control recombinant adenoviruses (ADGFP) (Fig. 4a,b,c). Similar changes in Runx2 and OPN expression were also observed at the protein level as indicated by western blotting (Fig. 4d,e).

### Inhibition of osteogenic differentiation by miR-1827 partially depends on IGF1

**Figure 5:**
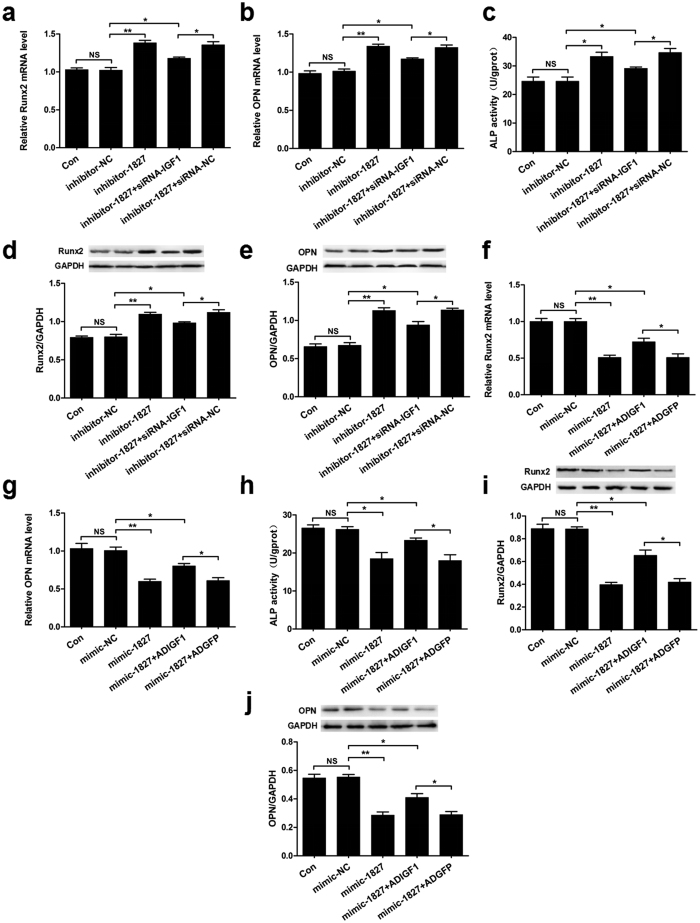
Inhibition of osteogenic differentiation by miR-1827 partially depends on IGF1. (**a**,**b**) qRT-PCR analysis of Runx2 and OPN mRNA expression in MSMSCs after co-transfection with miR-1827 inhibitor and siRNA-IGF1 or their respective negative controls. (**c**) Analysis of ALP activity in MSMSCs after co-transfection with miR-1827 inhibitor and siRNA-IGF1 or their respective negative controls. (**d,e**) Western blot analysis of Runx2 and OPN protein expression in MSMSCs after co-transfection with miR-1827 inhibitor and siRNA-IGF1 or their respective negative controls. (**f,g**) qRT-PCR analysis of Runx2 and OPN mRNA expression in MSMSCs after co-transfection with miR-1827 mimic and ADIGF1 or their respective negative controls. (**h**) Analysis of ALP activity in MSMSCs after co-transfection with miR-1827 mimic and ADIGF1 or their respective negative controls. (**i,j**) Western blot analysis of Runx2 and OPN protein expression in MSMSCs after co-transfection with miR-1827 mimic and ADIGF1 or their respective negative controls. For each group, values are the mean ± SD; n = 3, *P < 0.05, **P < 0.01. NS, not significant.

To further confirm that the inhibition of osteogenic differentiation by miR-1827 depends on IGF1, we co-transfected MSMSCs with the miR-1827 inhibitor and siRNA-IGF1 or their respective negative controls. The co-transfection of miR-1827 inhibitor with siRNA-IGF1 partially attenuated the miR-1827 inhibitor-induced increase in Runx2 and OPN mRNA expression as indicated by qRT-PCR (Fig. 5a,b). The increase in ALP activity induced by miR-1827 inhibitor was also partially inhibited (Fig. 5c). These changes persisted at the protein level, where co-transfection of miR-1827 inhibitor with siRNA-IGF1 partially blocked the increase in Runx2 and OPN protein expression induced by the miR-1827 inhibitor (Fig. 5d,e). Moreover, we transfected miR-1827 mimic into cells infected with ADIGF1 or ADGFP. The co-transfection of miR-1827 mimic with ADIGF1 partially blocked the miR-1827 mimic-induced reduction of Runx2 and OPN mRNA expression (Fig. 5f,g). The reduction of ALP activity induced by the miR-1827 inhibitor was also partially blocked (Fig. 5h). Similar alterations in Runx2 and OPN expression were also observed at the protein level (Fig. 5i,j).

### miR-1827 suppressed *in vivo* ectopic bone formation and the expression of IGF1 by human MSMSCs

**Figure 6:**
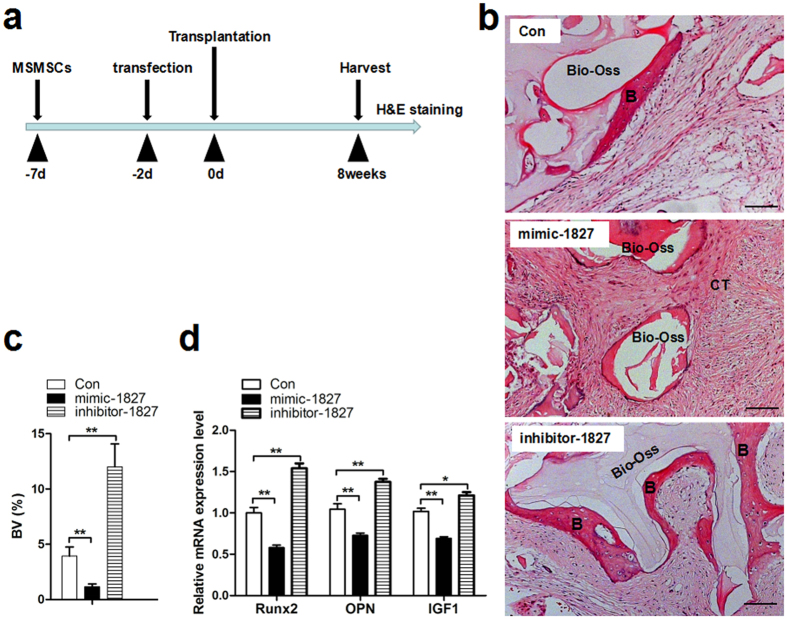
miR-1827 suppresses *in vivo* ectopic bone formation and the expression of IGF1 by human MSMSCs. (**a**) Scheme for assessing human MSMSCs-based ectopic bone formation. Human MSMSCs were amplified, transfected with miR-1827 mimic, inhibitor or their control and incubated for two days. The cells were then combined with Bio-Oss and transplanted into immunocompromised mice. After eight weeks, the transplanted specimens were harvested, decalcified, embedded and stained with H&E. (**b**) Representative image from each sample. B, bone; CT, connective tissue. (**c**) Bone volume (BV) was quantified using Image Pro 5.0 system. Bone volume = new bone area /total section size. (**d**) qRT-PCR analysis of IGF1 and osteogenic-specific markers (Runx2 and OPN) expression in MSMSCs transplant. For each group, values are the mean ± SD; n = 8, *P < 0.05, **P < 0.01. NS, not significant. Scar bars represent 100 μm.

Because the results described above demonstrated that miR-1827 exerts a negative regulatory effect on the osteogenic differentiation of MSMSCs *in vitro*, we next explored whether the regulation of the miR-1827 expression levels in MSMSCs also exerts an effect on bone formation *in vivo.* To induce ectopic bone formation, human MSMSCs were transplanted in conjunction with Bio-Oss scaffolds into immunocompromised mice, as depicted in Fig. 6a. Eight weeks after transplantation, new bone formation were also observed. Compared with the miR-1827 control-transfected MSMSCs transplant, the miR-1827 mimic-transfected MSMSCs showed significantly suppressed ectopic bone formation (Fig. 6b,c), whereas miR-1827 inhibitor enhanced ectopic bone formation (Fig. 6b,c). Moreover, compared with miR-1827 control-treated MSMSCs transplant, the mRNA expression levels of IGF1 and osteogenic-specific markers (Runx2 and OPN) appeared to be lower in miR-1827 mimic-treated MSMSCs transplant (Fig. 6d). On the contrary, the mRNA expression levels of IGF1 and osteogenic-specific markers (Runx2 and OPN) appeared to be higher in miR-1827 inhibitor-treated MSMSCs transplant (Fig. 6d). These results indicated that miR-1827 suppresses ectopic bone formation and the mRNA expression of IGF1 *in vivo*.

### Silencing of miR-1827 led to increased bone formation *in vivo*

**Figure 7:**
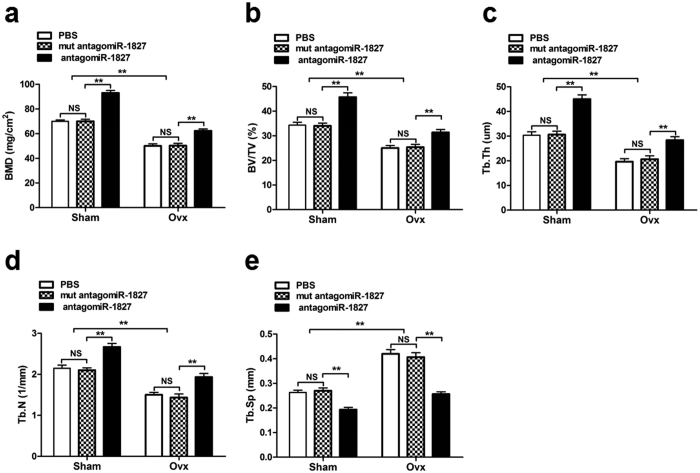
Silencing of miR-1827 led to increased bone formation *in vivo*. To investigate the function of miR-1827 *in vivo*, sham or OVX mice were injected i.v. with antagomiR-1827, mut antagomiR-1827, or PBS and bones were harvested at six weeks after the first injection. (**a**) BMD of femora was measured using a PIXImus densitometer. (**b,c,d,e**) Bone parameters of left femora including BV/TV, Tb.Th, Tb.N, and Tb.Sp were measured by micro-CT scanning using the GE explore Locus SP system. For each group, values are the mean ± SD; n = 8, **P < 0.01. NS, not significant.

To further investigate the function of miR-1827 *in vivo*, we performed an experiment in which a chemically modified antisense oligonucleotide specific to miR-1827. *In vivo* ready antagomiR-1827 was injected via a single tail vein injection into mice that had undergone sham operation or ovariectomy (Ovx). Mut antagomiR-1827 and PBS were used as controls. Silencing of miR-1827 by treatment of sham mice with antagomiR-1827 resulted in increase in bone mineral density (BMD) of left femora compared with the mut antagomiR-1827 or PBS control (Fig. 7a), Ovx mice treated with antagomiR-1827 also exhibited a significant increase in BMD (Fig. 7a). In addition, Quantification of micro-CT data revealed that antagomiR-1827-treated sham mice show significantly increased bone parameters of left femora, including bone volume/tissue volume ratio (BV/TV), trabecular thickness (Tb.Th) and trabecular number (Tb.N), with a concomitant decrease in trabecular spacing (Tb.Sp) (Fig. 7b,c,d,e), Ovx mice treated with antagomiR-1827 also exhibited a significant increase in BV/TV, Tb.N and Tb.Th and decrease in Tb.Sp (Fig. 7b,c,d,e).These results indicated that Silencing of miR-1827 led to increased bone formation and rescues bone insufficiency *in vivo*.

## Discussion

In the present study, we observed that MSMSCs expressed several different miRNAs, some of which were highly expressed after osteogenic differentiation. In addition, miR-1827 expression varied similarly in BMSSCs. Here, we focused on miR-1827 and sought to determine whether this molecule could be used *in vitro* to modulate osteogenic differentiation. Reintroduction of miR-1827 significantly inhibited the differentiation of MSMSCs. miR-1827 regulated osteogenic differentiation by repressing IGF1 expression at the transcriptional levels. Moreover, we demonstrated that IGF1 positively regulates osteogenic differentiation. Further study showed that miR-1827 suppressed *in vivo* ectopic bone formation and the expression of IGF1 by human MSMSCs. Silencing of miR-1827 led to increased bone formation *in vivo.* To our knowledge, this study shows for the first time that miR-1827 acts as a key regulator of osteogenic differentiation.

miR-1827 was recently reported to target L-MYC, and a nucleotide polymorphism for the miR-1827 binding site in the L-MYC3′-UTR is associated with an increased risk for lung cancer, indicating an important role for miR-1827 in suppressing lung cancer^18^. The level of circulating miR-1827 in serum was also found to be decreased in ulcerative colitis patients, who have an increased risk for colorectal cancer^21^. Further work indicated a novel role for miR-1827 indirectly targeting mouse double minute 2 (MDM2) to regulate p53, which in turn suppresses colorectal tumorigenesis^17^. All of these studies show a comprehensive and complex regulatory role for miR-1827 in cancer. However, the roles of miR-1827 in osteogenic differentiation, especially the molecular mechanisms we described in this study, have not been previously reported.

Runx2 is a key transcription factor in the commitment of multipotent mesenchymal cells to the osteogenic lineage and serves as a regulator of osteogenic differentiation^22^. ALP activity is always used as an indicator of osteogenic differentiation. OPN is a non-collagenous bone matrix protein and marks the late stages of osteogenic differentiation^23^. In this study, we identified that the expression levels of miR-1827 were up-regulated during the osteogenic differentiation of MSMSCs and that miR-1827 regulates osteogenic differentiation in MSMSCs. Over expression of miR-1827 suppressed ALP activity and the expression of canonical biomarkers of osteogenic differentiation (i.e., Runx2and OPN). Moreover, the formation of mineralized nodules was also reduced. On the other hand, inhibition of endogenous miR-1827 promoted ALP activity and induced the expression of osteogenic biomarkers. Our results showed that miR-1827 is up-regulated during the osteogenic differentiation of MSMSCs, and its expression is negatively associated with osteogenic differentiation. Moreover, the expression of miR-1827 was significantly up-regulated in differentiated BMSSCs and inhibited BMSSC osteogenic differentiation. These data indicate that miR-1827 may act as a common negative regulator of osteogenic differentiation. The increased miR-1827 expression in differentiated cells may stabilize the cell phenotype and reduce responsiveness to differentiation stimuli.

To address the molecular mechanisms by which miR-1827 regulates osteogenic differentiation, we predicted the potential targets of miR-1827 using TargetScan, miRanda and miRDB software. Notably, the 3′-UTR of IGF1 possesses two 7-nt sequences that perfectly match the miR-1827 seed region. Our experimental data demonstrated that IGF1 is a target gene of miR-1827 in MSMSCs and BMSSCs, as indicated by a luciferase assay. Furthermore, miR-1827 negatively regulated IGF1 expression at the mRNA levels in MSMSCs.

The actions of IGF are important for normal bone growth and exert positive effects on bone density, bone size, and bone formation in mammals^24^,^25^,^26^. Specifically, IGF1 promotes bone homeostasis and development and has been shown to stimulate osteogenic proliferation and differentiation^19^,^27^,^28^,^29^,^30^. IGF1 mediates its effects by binding to IGF1 receptors on the cell membrane, activating the irintrinsic tyrosinekinase activity and enabling internalization of the receptor ligand complex to initiate signaling cascades with numerous biological effects^31^,^32^. Inhibition of the IGF1 receptor during osteogenesis causes impaired bone formation and reduced mineralization^33^. In the present study, our experiments confirmed that IGF1 could stimulate the osteogenic differentiation of MSMSCs, as indicated by strong expression of several osteogenic-specific markers. In addition, inhibition of osteogenic differentiation by miR-1827 partially depends on the repression of IGF1. IGF1 was identified as a downstream regulator of miR-1827 that participates in the mechanism by which miR-1827 regulates osteogenic differentiation. The increased miR-1827 expression during osteogenic differentiation likely acts as a negative feedback mechanism.

As miR-1827 exerts a negative regulatory effect on the osteogenic differentiation of MSMSCs *in vitro,* we next explored whether the regulation of the miR-1827 expression levels in MSMSCs also exerts an effect on bone formation *in vivo.* We found that miR-1827 suppressed *in vivo* ectopic bone formation in nude mice. Moreover, miR-1827 suppressed the expression of IGF1 by human MSMSCs, which is a target of miR-1827. Further study showed that silencing of miR-1827 led to an increase in bone mineral density in sham mice and enhanced bone mineral density gain in Ovx mice. Silencing of miR-1827 also led to significant improvement in trabecular microarchitecture in Ovx mice. In fact, silencing of miR-1827 led to an even enhanced effect on the trabecular architecture in sham mice. These data suggest that miR-1827 inhibits bone formation. Silencing of miR-1827 rescues bone insufficiency.

To our knowledge, this study is the first report demonstrating that miR-1827 serves as a negative regulator of osteogenic differentiation. Specifically, miR-1827 functions by inhibiting its direct target IGF1 at the transcriptional level to negatively regulate osteogenic differentiation. miR-1827 suppressed ectopic bone formation and silencing of miR-1827 led to increased bone formation *in vivo.* Our findings revealed a new function for miR-1827 and suggest that therapeutic approaches through inhibiting miR-1827 may be useful for regeneration of the atrophic posterior maxilla.

## Materials and Methods

### Ethics statement

All protocols and the informed consent form for MSM isolation were approved by the Sun Yat-Sen University Joint Institutional Review Board and performed in accordance with the guidelines of the Medical Ethics Committee of Sun Yat-Sen University. The specimen donors were provided the IRB-approved formal consent form describing sufficient information for one to make an informed decision about his/ her participation in this study. The formal consent forms were signed by the subjects before specimen collection.

### Samples and cell culture

Normal human MSM samples were obtained as previously reported^6^. MSMSCs were isolated and cultured as previously reported^6^. BMSSCs were obtained from ScienCell Research Laboratory (San Diego, CA, USA). All primary cells used in this study were passaged 2–4 times. For each experiment, the same passage of MSMSCs and BMSSCs was used.

### Cell differentiation

For osteogenic differentiation, MSMSCs and BMSSCs were cultured at a density of 1 × 10^4^ cells/cm^2^ in osteogenic medium containing DMEM, 10% FBS, 0.1 mM dexamethasone, 10 mM b-glycerophosphate, and 50 μg/mL ascorbicacid. The medium was changed every 72 h until analysis.

### Microarray analysis of miRNA expression

Total mRNA was extracted using the mirVana™ RNA Isolation Kit (Ambion, Foster City, CA, USA). miRNA expression profiles were determined by microarray analysis using the μ Paraflo™ microfluidic chip (MiHuman_8.2- Based on Sanger miRBase Release 8.2, LC Sciences) according to the manufacturer’s instructions.

### Quantitative RT-PCR

Total RNA isolation, first-strand cDNA synthesis, and PCR were performed as previously described^34^. The GAPDH gene was used as a standard control. U6 was employed for miRNA template normalization. The primers used for amplification are listed in Table 1.

### Cell transfection

To transfect cells with miRNA regulators and siRNA oligos, the medium was supplemented with Lipofectamine™ 2000 (Invitrogen, Carlsbad, CA, USA) according to the manufacturer’s instructions. Cells were transfected with miR-1827 mimic or inhibitor (RiboBio, Guangzhou, China) at a concentration of 50 nM. A siRNA targeting IGF1 was also designed. The sequences of this siRNA and its negative control are listed in Table 1. The siRNA was transfected at a concentration of 50 nM.

### Alkaline phosphatase activity analysis

Cells were seeded into 6-well plates (Costar, Cambridge, MA, USA) at a density of 2 × 10^4^ cells/well. After the indicated number of days of culture in calcification medium, ALP activity was detected using an ALP assay kit (Jian Cheng Co., Nanjing, China) according to the manufacturer’s instructions. The amount of ALP in the cells was normalized to the total protein content.

### Western blotting

The primary antibodies used for western blot analyses included Runx2 (Abcam, Cambridge, CB4 0FW, UK) (1:500), OPN (Cell Signaling Technology, Danvers, MA, USA) (1:500), IGF1 (Abgent, San Diego, CA, USA) (1:500), and GAPDH (Cell Signaling Technology) (1:1000). Western blot analyses were performed as previously reported^2^.

### Alizarin red staining

Cells were seeded into 6-well plates (Costar) at a density of 2 × 10^4^ cells/well. After the indicated number of days of culture in calcification medium, mineralized matrix nodules were stained with Alizarin Red S (Sigma, St. Louis, MO, USA) as described previously^35^. Stained mineralized matrix nodules were scanned and/or imaged under a microscope. For Alizarin red quantification, 1 ml of 10% cetylpyridinium chloride (Sigma) was added to each well. Light absorbance of the extracted dye was measured at 562 nm.

### Luciferase assay

Cells were seeded into 96-well plates (Costar) at a density of 2 × 10^4^ cells/well. Using Lipofectamine 2000 (Invitrogen), cells were transfected with 100 ng of empty pRL-TKvector (Promega, Madison, WI, USA), pRL-TK-IGF1-WT 3′ UTR, or pRL-TK-IGF1-Mut 3′ UTR. Then, these cells were co-transfected with miR-1827 mimic, miR-1827 inhibitor or their respective negative controls at a concentration of 50 nM. Cells were harvested for the luciferase assay 48 h after transfection using a luciferase assay kit (Promega) according to the manufacturer’s instructions.

### Construction of recombinant adenoviruses expressing IGF1

Recombinant adenoviruses were generated using AdEasy technology as previously described^36^. The coding sequence of human IGF1 was amplified using PCR, cloned into an adenoviral shuttle vector, and subsequently used to generate recombinant adenoviruses in HEK293 cells. The recombinant adenoviruses were designated as AdIGF1. AdIGF1 was then tagged with green fluorescent protein (GFP) to track infected cells. Analogous adenoviruses expressing only monomeric GFP (AdGFP) were used as controls.

### Transplantation

The animal study was approved by the Ethical Committee on Animal Research of the Sun Yat-Sen University. All experimental procedures were performed according to national guidelines regarding the care and use of laboratory animals. Specific-pathogen-free (SPF) male immunocompromised mice (5-week-old, BALB/c-nu) (n = 8 for each group) were purchased from the Experimental Animal Department of the Chinese Academy of Sciences, and were maintained in SPF conditions in the whole process of experiment. Human MSMSCs were transfected with 80 nM miR-1827 mimic, inhibitor or their control two days before transplantation. Approximately 4 × 10^6^ *in vitro*- expanded MSMSCs were combined with 40 mg of deproteinized bovine bone (Bio-Oss; Geistlich, Wolhusen, Switzerland) and then transplanted into dorsal subcutaneous pockets, as described in previous studies^37^. Briefly, the mice were anesthetized. The midline surgical incisions of approximately 2 cm in length were made on the back of mice. Blunt dissection away from the midline cranially and caudally to the left and the right of the spine followed to form 3 subcutaneous pockets. Each animal received three randomly allocated experimental materials of the following groups: i) miR-1827 mimic, ii) miR-1827 inhibitor and iii) control group. The experimental materials were implanted in such a way that experimental materials from all the experimental groups are implanted in all possible locations among following three sites: upper right side, upper left side and lower left side. All transplants were harvested after eight weeks and divided into two equal halves for further analysis. One half of the transplant fragments was stored for total RNA extraction and the relative expressions of the genes encoding Runx2, OPN, IGF1, and GAPDH were evaluated by qRT-PCR. The other half was fixed with 10% buffered formalin and then decalcified with 10% edetic acid (pH 8.0), for two weeks. The decalcified transplants were embedded in paraffin, sectioned to a thickness of 3 μm, and stained with haematoxylin and eosin (H&E). The percentage of new bone area per total section area was calculated using the average value of the three randomly selected parallel slices with Image Pro 5.0 system (Media Cybernetics, Silver Springs, MD, USA). The mean value of the three measurements was calculated for each transplant and was further used to calculate mean values for each group.

### Mice bone insufficiency model

Specific-pathogen-free (SPF) female mice (6-week-old, BALB/c) were purchased from the Experimental Animal Department of the Chinese Academy of Sciences, and were maintained in SPF conditions in the whole process of experiment. These animals had free access to autoclaved water and a pellet diet for one week prior to the surgery. Next, a sham-operation was performed on the mice or the mice were surgically ovariectomized after anaesthesia by pentobarbital sodium. The ovariectomy operation was performed as previously reported^38^. These mice received antagomiR-1827 (Life Technologies, Carlsbad, CA, USA) or mut antagomiR-1827 for 3 consecutive days in the first week followed by another injection on days 1–3 of the fourth week, at a dose of 7 mg/kg body weight or a comparable volume of PBS (0.2 ml) through tail vein injection^39^. Six weeks after the first injection, mice were euthanized and bones were harvested. The left femur of each mouse was fixed onto the scanning table along the longitudinal axis, and the whole femur was scanned by DXA using a PIXImus densitometer (GE Healthcare, Madison, Wisconsin, USA) to determine the BMD. The femur of each mouse was then fixed with 4% paraformaldehyde for 24 h and subsequently washed with 10% saccharose solution; 12 h later, Bone parameters of left femora including BV/TV, Tb.Th, Tb.N, and Tb.Sp were measured by micro-CT scanning using the GE explore Locus SP system (GE Healthcare).

### Statistical analysis

Each experiment was performed in triplicate and repeated at least three times *in vitro.* 8 mice in each group were used *in vivo*. The experimental data were statistically analyzed with SPSS 17.0 software (SPSS, Chicago, IL, USA). The data are expressed as the mean ± SD. A repeated-measures one-way ANOVA was used to compare the time-course variables. Comparisons were performed using a two-tailed t-test or one-way ANOVA for experiments with more than two subgroups. All P-values are two-tailed, and P < 0.05 was considered statistically significant.

## Additional Information

**How to cite this article**: Zhu, S.X. *et al*. miR-1827 inhibits osteogenic differentiation by targeting IGF1 in MSMSCs. *Sci. Rep.* **7**, 46136; doi: 10.1038/srep46136 (2017).

**Publisher's note:** Springer Nature remains neutral with regard to jurisdictional claims in published maps and institutional affiliations.

## Supplementary Material

Supplementary Information

## Figures and Tables

**Table 1:** The sequence of primers and siRNAs.

Primer	Sequences(5′-3′)
miR-1827 RT	GTCGTATCCAGTGCAGGGTCCGAGGTATTCGCACTGGATACGACATTCAA
miR-1827- F	GGGGTGAGGCAGTAGATTG
miR-20b RT	GTCGTATCCAGTGCAGGGTCCGAGGTATTCGCACTGGATACGACCTGGAA
miR-20b- F	GGAACTGTAGTATGGGCACTT
miR-124 RT	GTCGTATCCAGTGCAGGGTCCGAGGTATTCGCACTGGATACGACGGCATT
miR-124- F	TCTAAGGCACGCGGTGAA
miR-186a-5p RT	GTCGTATCCAGTGCAGGGTCCGAGGTATTCGCACTGGATACGACAGCCCA
miR-186a-5p-F	GCCCCAAAGAATTCTCCTTTTG
miR-18a RT	GTCGTATCCAGTGCAGGGTCCGAGGTATTCGCACTGGATACGACCCAGAA
miR-18a-F	ACTGCCCTAAGTGCTCCTT
miR-93 RT	GTCGTATCCAGTGCAGGGTCCGAGGTATTCGCACTGGATACGACCGGGAA
miR-93-F	CACTGCTGAGCTAGCACTT
miR-34b RT	GTCGTATCCAGTGCAGGGTCCGAGGTATTCGCACTGGATACGACATGGCA
miR-34b-F	CCCCAATCACTAACTCCACTG
miR-140 RT	GTCGTATCCAGTGCAGGGTCCGAGGTATTCGCACTGGATACGACCCGTGG
miR-140-F	GATGTACCACAGGGTAGAACC
miR-224-5p RT	GTCGTATCCAGTGCAGGGTCCGAGGTATTCGCACTGGATACGACAACGGA
miR-224-5p-F	GGGCAAGTCACTAGTGGTTC
miR-27a RT	GTCGTATCCAGTGCAGGGTCCGAGGTATTCGCACTGGATACGACGCGGAA
miR-27a-F	CCCTTCACAGTGGCTAAGTT
all miR reverse primer	GTGCAGGGTCCGAGGT
U6-F	CTCGCTTCGGCAGCACA
U6-R	AACGCTTCACGAATTTGCGT
Runx2-F	CACTGGCGCTGCAACAAGA
Runx2-R	CATTCCGG-AGCTCAGCAGAATAA
OPN-F	GCCGACCAAGGAAAACTCACT
OPN-R	GGCACAGGTGATGCCTAGGA
IGF1-F	GGAGCTGTGATCTAAGGAGGC
IGF1-R	GGGCTGATACTTCTGGGTCTT
GAPDH-F	AGCCGCATCTTCTTTTGCGTC
GAPDH-R	TCATATTTGGCAGGTTTTTCT
siRNA-IGF1 sense	GGAGUGCAGGAAACAAGAATT
siRNA-IGF1 antisense	UUCUUGUUUCCUGCACUCCTT
siRNA-NC sense	UUCUCCGAACGUGUCACGUT
siRNA-NC antisense	ACGUGACACGUUCGGAGAA
