# Supplementary material for: miR-1827 inhibits osteogenic differentiation by targeting IGF1 in MSMSCs
Source: Sci Rep. 2017 Apr 7;7:46136. doi: 10.1038/srep46136 (PMC5384002; doi:10.1038/srep46136)
Supplement: Supplementary Information [file srep46136-s1.pdf]

Supplementary Information

miR-1827 inhibits osteogenic differentiation by targeting IGF1 in MSMSCs

ShuangXi Zhu[a,b,1], Wei Peng[a,b,1], Xiang Li[a,b], JunQuan Weng[a,b], Xing Zhang[c], JunBing Guo[a,b], DaiYing Huang[a,b], Qiong Rong[d], SongLing Chen[a,b,*]

Supplementary Figure 1

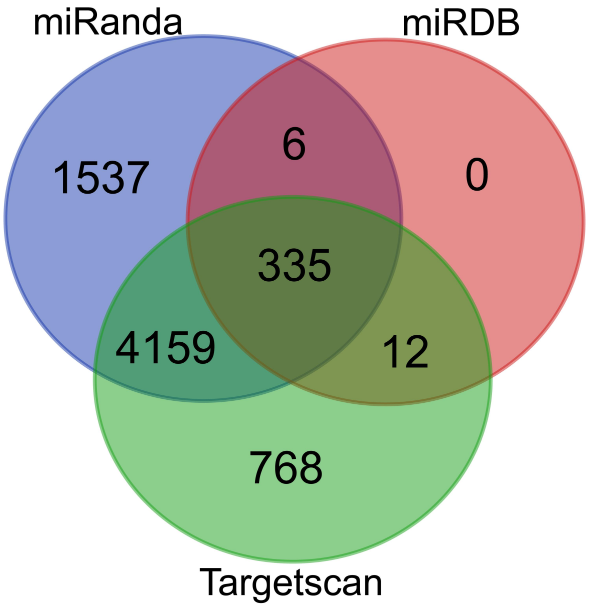

Supplementary Figure 1. Potential target genes of miR-1827, predicted using TargetScan, miRanda and miRDB software. In order to minimize possible false positive prediction, only genes predicted by three software were identified as potential target genes of miR-1827. Numbers refer to genes predicted by one of the three software programs.

Supplementary Figure 2

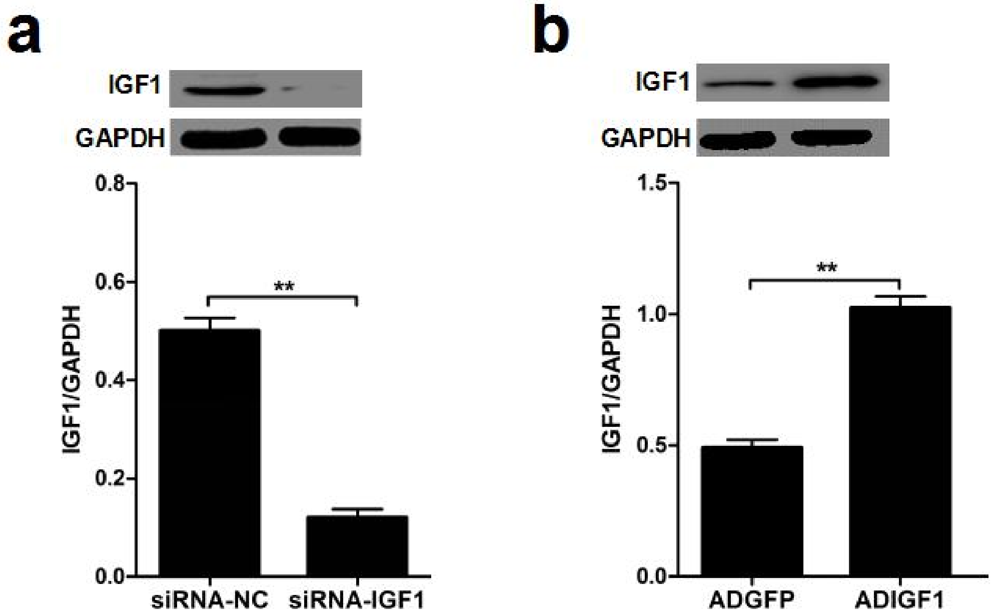

Supplementary Figure 2. The regulative effect of siRNA-IGF1 and ADIGF on IGF1 protein. (**a**) Western blot analysis of the changes in IGF1 protein expression in MSMSCs after transfection with siRNA-IGF1 or its negative control for 2d. (50 nM). (**b**) Western blot analysis of the changes in IGF1 protein expression in MSMSCs after transfection with ADIGF1 or ADGFP for 2d. (200 ng/μl). For each group, values are the mean ± SD; n = 3, ***P* <0.01. NS, not significant.
